# sRNAtoolbox: an integrated collection of small RNA research tools

**DOI:** 10.1093/nar/gkv555

**Published:** 2015-05-27

**Authors:** Antonio Rueda, Guillermo Barturen, Ricardo Lebrón, Cristina Gómez-Martín, Ángel Alganza, José L. Oliver, Michael Hackenberg

**Affiliations:** 1 Genomics and Bioinformatics Platform of Andalusia (GBPA), Edificio INSUR, Calle Albert Einstein, 41092-Sevilla, Spain; 2 Centro de Genómica e Investigaciones Oncológicas, Pfizer-Universidad de Granada-Junta de Andalucía, Granada 18016, Spain; 3 Dpto. de Genética, Facultad de Ciencias, Universidad de Granada, Campus de Fuentenueva s/n, 18071-Granada, Spain; 4 Lab. de Bioinformática, Centro de Investigación Biomédica, PTS, Avda. del Conocimiento s/n, 18100-Granada, Spain

## Abstract

Small RNA research is a rapidly growing field. Apart from microRNAs, which are important regulators of gene expression, other types of functional small RNA molecules have been reported in animals and plants. MicroRNAs are important in host-microbe interactions and parasite microRNAs might modulate the innate immunity of the host. Furthermore, small RNAs can be detected in bodily fluids making them attractive non-invasive biomarker candidates. Given the general broad interest in small RNAs, and in particular microRNAs, a large number of bioinformatics aided analysis types are needed by the scientific community. To facilitate integrated sRNA research, we developed sRNAtoolbox, a set of independent but interconnected tools for expression profiling from high-throughput sequencing data, consensus differential expression, target gene prediction, visual exploration in a genome context as a function of read length, gene list analysis and blast search of unmapped reads. All tools can be used independently or for the exploration and downstream analysis of sRNAbench results. Workflows like the prediction of consensus target genes of parasite microRNAs in the host followed by the detection of enriched pathways can be easily established. The web-interface interconnecting all these tools is available at http://bioinfo5.ugr.es/srnatoolbox

## INTRODUCTION

Over the last years, high-throughput sequencing (HTS) technologies changed notably our understanding of the biogenesis and function of small RNA molecules. Apart from microRNAs, which are known for over a decade to play very important roles in gene regulation (1,2), several other classes of small RNAs have been described. In plants, small interfering RNAs (siRNA), trans-acting RNAs and heterochromatic siRNAs are involved in the regulation of gene expression and the chromatin state by depositing repressive marks (DNA methylation and histone marks) (3). Furthermore, they seem to play important roles in abiotic stress response in plants (4,5). In animals, reproducibly processed small RNA fragments of yRNA, tRNA and snoRNA origin have been identified (6) and especially tRNA fragments might be functional by regulating translation (7).

Over the last years, several other research fields emerged in which microRNAs and other small non-coding RNAs play vital roles. MicroRNAs seem to be crucial in the evasion of the immune response and the regulation of the switch to lytic cycle of virus infections (8,9) and in general might have important functions in host-microbe interactions (10). Furthermore, microRNAs have strong biomarker potential as specific miRNA expression profiles have been associated with different types of cancer, tumour development and allowed to identify the tissue of origin of poorly differentiated tumours (11). Small RNAs are also present in membrane-bound vesicles called exosomes which are secreted by virtually all cell types (12,13). As a consequence, microRNAs can be detected in most studied bodily fluids and are therefore perfect candidates for non-invasive biomarkers (14). Finally, microRNAs have been also detected in exosomes secreted by parasites (15) being probably involved in the modulation of the innate immunity of the host (16).

Given the wide-ranging interest in small RNAs, a broad panoply of bioinformatics tools is needed to assist researchers from different fields in small RNA research. Often the first step consists in the expression profiling of small RNAs by means of HTS approaches. Frequently used programs for this task are: small RNA toolkit (17), miRDeep (18), miRanalyzer (19,20), SeqBuster (21), DARIO (22), UEA sRNA workbench (23), miRDeep2 (24) and ShortStack (25). Those tools do generally also include other important aspects like the prediction of novel microRNAs or the isomiR quantification. However, they do generally not offer functional downstream analysis, visualization of the expression pattern in a genome context or the capacity to analyse multi-species assays. Some exceptions are mirTools 2.0 (26), which includes target prediction and functional analysis linked to the expression profiling, UEA sRNA workbench including visualization and sRNAbench (27) which allows the expression profiling in experiments that involve genetic material from more than one species.

Many important analysis types are currently not available, are linked to a fixed pipeline or are scattered over several independent tools. Given the increasing interest in studying microbe-host interactions, one frequently needed analysis type would be to estimate the impact of parasite secreted microRNAs on the host genes, i.e. which genes and which pathways might be regulated by those exogenous microRNAs. Another example could be the visual exploration of small RNA differential expression in a genome context as a function of read length, given that at least in plants 24 nt long reads have clearly different functions than 21/22 nt long reads.

In order to overcome some of the mentioned lack of analysis types, we developed sRNAtoolbox having in mind two main aims. First, we implemented some analysis types that are currently missing, like the cross-species microRNA target prediction coupled to functional *in silico* analysis, the consensus detection of differentially expressed sRNAs or the visualization of differential expression in a genome context as a function of read length. Second, we implemented workflows in order to make some analysis types easier, opening therefore the analysis to users with less bioinformatics backgrounds. The centre piece of sRNAtoolbox is sRNAbench that allows the expression profiling and prediction of novel microRNAs in a multi-species experimental assay. Most other tools, interconnected through the same web-interface, can be used for downstream and exploratory analyses on sRNAbench output, but also in an independent manner on the appropriate input. This makes sRNAtoolbox a very flexible and easy to use set of small RNA research tools.

## WORKFLOW AND SCOPE

sRNAtoolbox consists currently of seven independent but interrelated tools and one workflow addressing several important analysis types. The web-interface interconnecting all these tools is implemented in Django and javascript while the underlying programs are written in Java. The required data are stored either in mongoDB or in MySQL depending on the nature and structure of the data. The results will be stored for 15 days on the server.

Briefly, sRNAtoolbox contains the following tools:

- ***sRNAbench***: expression profiling of small RNAs, prediction of novel microRNAs, analysis of isomiRs, genome mapping and read length statistics. The samples are analysed individually.
- ***sRNAde***: detection of differentially expressed small RNAs based on three commonly used programs: DESeq (28), edgeR (29) and NOISeq (30). To our knowledge it is the first tool that determines a consensus of differentially expressed sRNA.
- ***sRNAblast***: aimed to determine the origin of unmapped or unassigned reads by means of a blast search against several remote databases. The results can either point towards contamination sources or biological meaningful information like the presence of unexpected viral or bacterial RNA molecules.
- ***miRNAconsTarget***: consensus target prediction on user provided input data based on Miranda (1), PITA (31) and TargetSpy (32) for animal microRNAs and PsRobot (33) and TAPIR (34) for plant microRNAs.
- ***sRNAjBrowser***: visualization of sRNA expression data in a genome context using jBrowse (35).
- ***sRNAjBrowserDE***: visualization of differential expression as a function of read length in a genome context using jBrowse.
- ***sRNAfuncTerms***: enrichment of functional annotations in animal and plant target genes not only for a set of microRNAs but also for all its combinations (microRNA modules).
- ***sRNAfuncTargets (workflow)***: cross-species microRNA target prediction and enrichment analysis of functional annotations.

**Figure 1.**
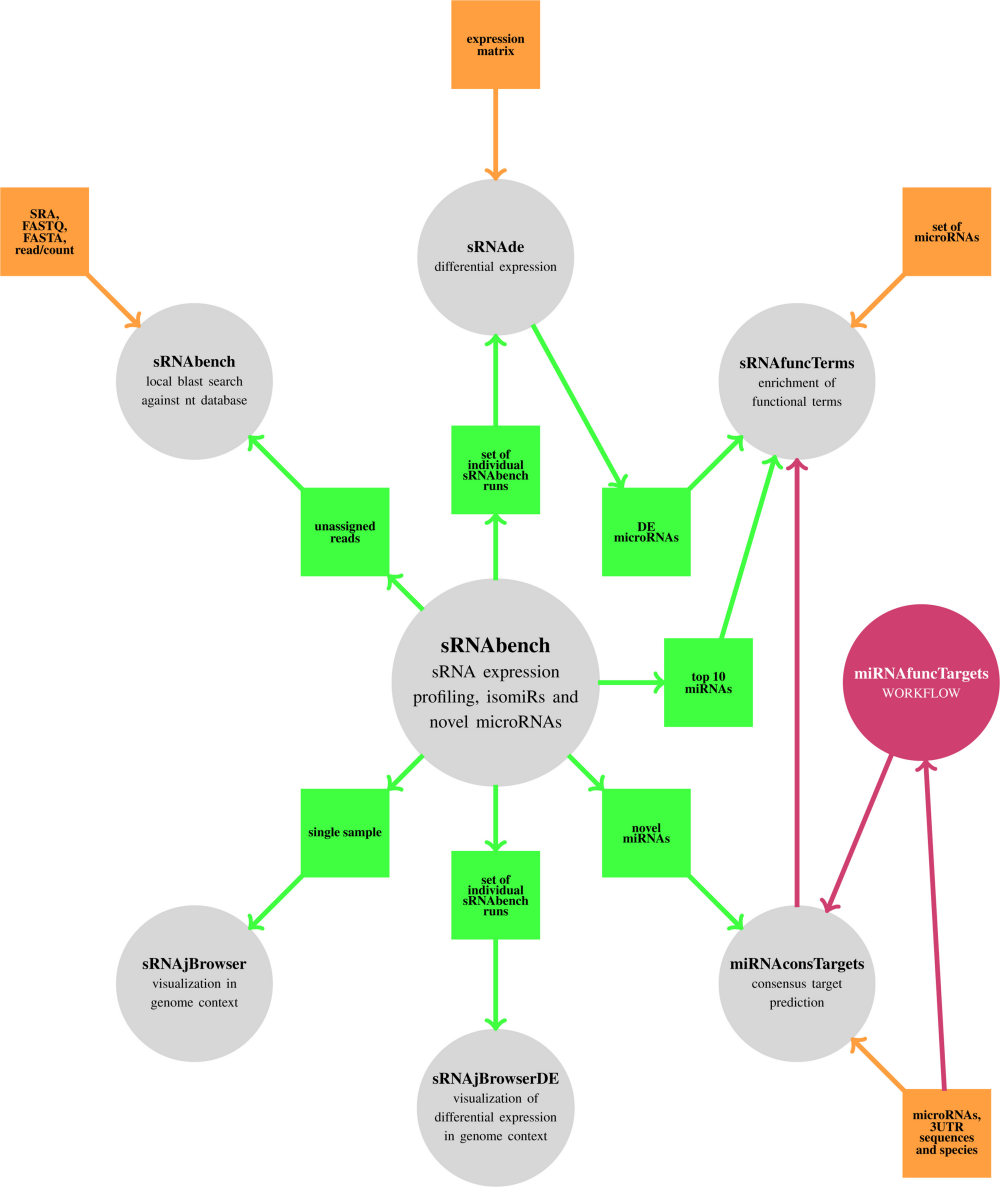
Relation between the different tools. The way the tools interact with sRNAbench (green) and how they can be used independently (orange) is depicted.

An important point in sRNAtoolbox is that although sRNAbench is the center-piece, most tools can be used also in an independent way. Figure 1 shows the relation between the different tools and mentions briefly the input types that can be used.

## DESCRIPTION OF THE INDIVIDUAL TOOLS

Given that sRNAbench was published before (27), in this paragraph we will describe primarily the other tools that comprise the toolbox and the way in which these tools are interconnected, connected to sRNAbench or in which way they can be used independently.

### sRNAde: consensus detection of differentially expressed sRNA

The sRNAde module can be used in two different ways. It can be applied either to a user-generated expression matrix or to a number of individual sRNAbench runs indicating the relation between the samples on the input, i.e. defining the experimental groups between which the differentially expressed sRNA should be detected. Independently of the input type, two main results are generated. First, heatmaps are calculated using Hierarchical Clustering (implementation: hclust for R, parameters method = ‘complete’) which allows the user to visualize the clustering of the samples and to detect outlier samples. Second, the differential expression is assessed using three frequently used programs: edgeR, DESeq and NOISeq. Additionally, the program also generates a consensus differential expression file which gives the user the possibility to increase the stringency using only those microRNAs/sRNA that have been detected as differentially expressed by more than one method.

If sRNAbench output is used as input, additional features are available. For example, the module generates a summary of sequencing, adapter removal and genome mapping statistics, both in table and graphic format. In this way, poor samples can be easily spotted and probably removed from the analysis.

### sRNAblast

Frequently it happens that only a minor fraction of the reads can be mapped to the genome or that a high fraction of the reads cannot be assigned to any annotation. Two basic explanations exist for unmapped reads: bad sequencing quality or contamination. While the first possibility can be assessed by means of tools like fastQC, the search for possible contamination sources is more daunting. sRNAblast uses a local installation of blast+ (36) and allows to detect the reads in several remote databases hosted at NCBI: nr (all GenBank, RefSeq Nucleotides, EMBL, DDBJ and PDB sequences), refseq_rna (RNA entries from NCBI's Reference Sequence project), est (Database of GenBank, EMBL, DDBJ sequences from EST Divisions) and env_nt (Nucleotide sequences from environmental samples) in order to aid the identification of unmapped/unassigned reads. Given that the mapping with blast of thousands of reads is a CPU consuming task, sRNAblast is mainly intended for the annotation of unmapped reads from a previous sRNAbench run. It can, however, also be used as an independent tool. It shares the sRNAbench preprocessing and accepts therefore fastq, sra, fasta and read/count format and is able to perform adapter trimming.

### miRNAconsTargets: consensus microRNA target prediction

Currently many of the most frequently used microRNA target prediction programs are available over web-server programs or databases and do therefore not allow a user customized prediction of microRNA targets. miRNAconsTargets allows the user to upload a set of target sequences or choosing a 3’ UTR set from our database together with a set of microRNAs. TargetSpy, Miranda and PITA are available for the prediction of animal microRNA targets and PsRobot and TAPIR are used for plants. The target prediction pipelines generate both, individual predictions for each program and a more reliable consensus prediction. Furthermore, the parameters of the different programs can be changed by the user making the consensus target prediction very flexible.

### sRNAfuncTerms

One of the first questions after having obtained a number of differentially expressed microRNAs or even a set of highly expressed microRNAs in a single sample is on its putative functions. A generally applied approach consists of determining the target genes and performing an enrichment/depletion analysis of functional annotations on this gene list. This can give important insight into the molecular functions or biological processes of the regulated genes.

sRNAfuncTerms uses Gene Ontology as functional annotations (37) and is based on AnnotationModules (38) to extract those functional annotations that are enriched among the target genes in a statistically significant way.

sRNAfuncTerms can be used on sRNAbench, sRNAde results and independently by providing microRNA names and the species. For sRNAbench, the 10 most expressed microRNAs are analysed while for sRNAde input the statistically significant differentially expressed microRNAs detected by any of the methods are analysed.

In a first step, the target genes are assigned by means of an underlying database which holds experimentally verified targets from TarBase v6 (39), predictions obtained by means of the psRNAtarget web-server for plants (40) and predictions using the miRNAconsTargets pipeline explained in the last section (both for plants and animals). The frequency of each functional annotation in the list of target genes is then compared to the background of all annotated genes by means of the local implementation of the AnnotationModules tool.

As a novel feature, sRNAfuncTerms does not only determine the enriched annotations for the complete set of microRNAs, but also for microRNA modules up to a certain depth or module size.

Briefly, sRNAfuncTerms determines the enrichment for all individual microRNAs, and all combinations (or modules) of two and three microRNAs. If a functional annotation is detected for more than one microRNA module, the one with the highest enrichment ratio is reported. In this way, the researcher obtains the knowledge on which concrete microRNAs might be involved in a certain pathway or function.

### sRNAfuncTargets

sRNAconsTargets and sRNAfuncTerms can be used directly as a pipeline. This enables prediction of the target genes of a set of microRNAs (independently of the origin) in any of the species included in sRNAtoolbox (either using our database or user provided target sequences). The consensus target genes are then used by sRNAfuncTerms to determine the enriched functional annotations. This allows the user to determine which genes and pathways might be regulated, for example by parasite microRNAs within a host cell. Please note that cross-species target prediction can be carried out also by the psRNAtarget server for plants, however without a functional downstream analysis.

### sRNAjBrowser and sRNAjBrowserDE

The visualization of expression values in a genome context can give important information. For example in virus research, the mapping profile can indicate the existence of a previously unknown small non-coding RNA. Both tools share the basic workflow, but while sRNAjBrowser visualizes the expression values reads per million (RPM) of a single sample, sRNAjBrowserDE depicts the differential expression values together with the logarithm of the mean expression values of each experimental group.

In general, the tools are based on the genome mappings that sRNAbench generates by means of the Bowtie aligner (41). In a first step, the mappings are converted into internal bedGraph files. Reads that map to several positions in the genome are fully assigned to each locus, i.e. by default, no proportional assignment is applied. The number of mapped reads per position is then converted into RPM using the total number of genome mapped reads for normalization. By means of the bedGraphToBigWig tool provided by UCSC, the bedGraph files are then converted into bigwig format which can be visualized by means of the implemented jBrowse (35).

The sRNAjBrowserDE tool allows furthermore the analysis as a function of read length. It sums first the mappings of all samples of one condition before assigning a RPM value to each position. In a second step, the log2 of the fold-change is calculated for each position. In order to avoid division by zero, a fixed value of 10 is added to each RPM before applying the log2 to the case/control ratio (fold-change).

The bigwig files can then be visualized together with several genome annotation tracks including microRNA (both mature and precursor sequences), genes and repetitive elements.

## WORKING EXAMPLES

**Figure 2.**
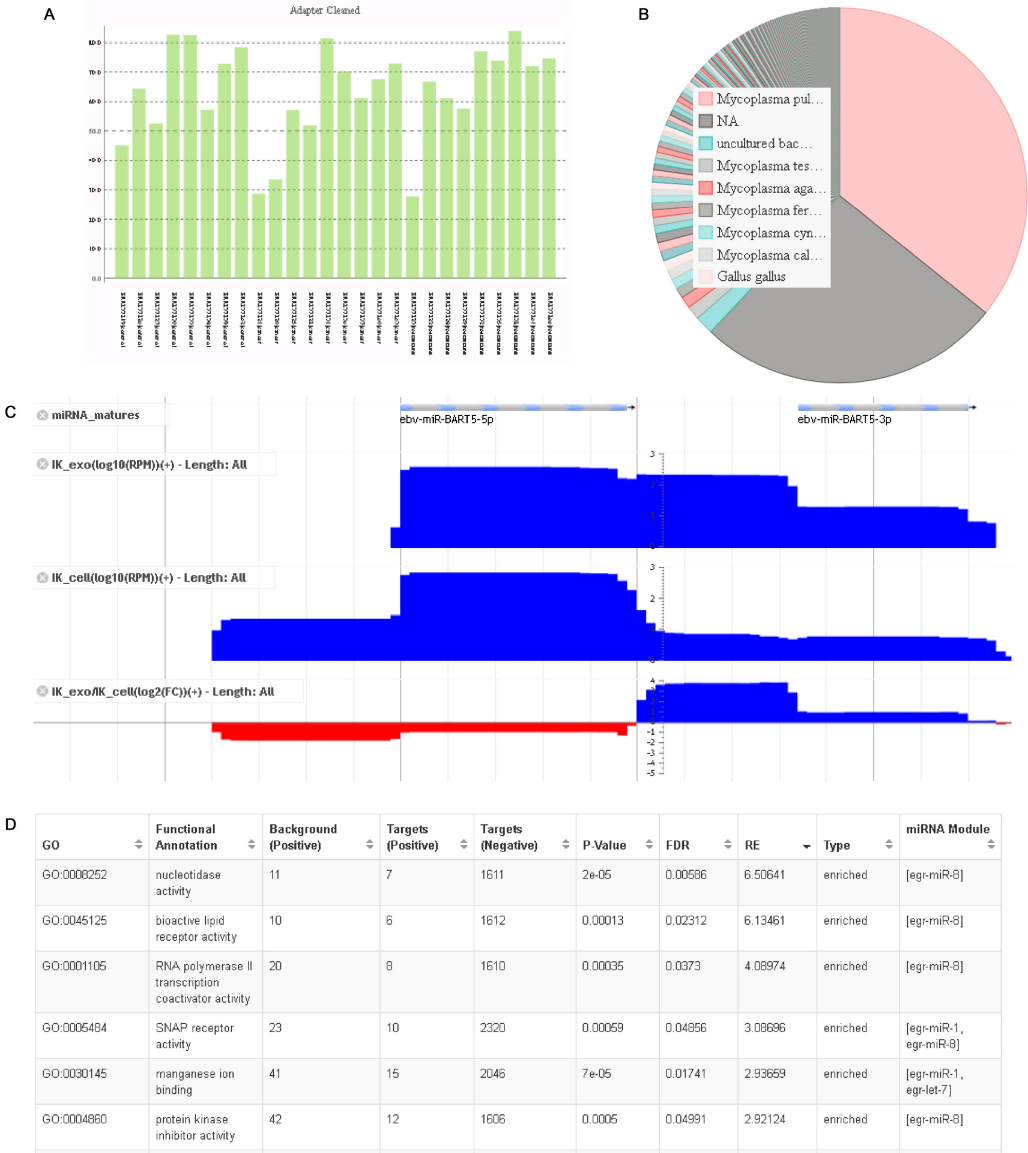
Results for four different working examples. (**A**) Percentage of adapter trimmed reads in 26 samples of colorectal cancer; (**B**) graphical representation of the blast search of reads not mapped to the human genome from a SRA data set (SRR1057370); (**C**) differential expression between small RNAs in cells and exosomes from a LCL cell line (IK). The bottom line shows the log_2_ of the fold-change indicating that the most differentially expressed region is the loop region of viral microRNA mir-BART5; (**D**) a screen-shot of the most enriched molecular functions among the cow target genes of three *E. granulosus* microRNAs.

Figure 2 summarizes the results of four working examples in order to demonstrate the usefulness of sRNAtoolbox. Figure 2A depicts the summary of adapter cleaning of 26 samples for colorectal cancer (42) which is used as test data set for sRNAde. The sRNAde tool gives several summary graphics at an experiment level which can help the user to easily detect problematic samples. In this case, three samples can be seen with only around 30% of detected adapters while on average this figure is around 70% in the whole experiment.

To show the usefulness of sRNAblast we reanalysed a publicly available data set of *Salmonella enteritidis* (strain SE2472) infected HT-29 cells (43). After processing the data (SRR1057370) with sRNAbench, we analysed the unmapped reads with sRNAblast. Figure 2B shows that most unmapped reads were assigned to *Mycoplasma pulmonis* and not to *Salmonella enteritidis*. Figure 2C shows an example of differential expression between an Epstein–Barr virus (EBV) infected Lymphoblastoid Cell Line (LCL cell line) and its secreted exosomes (35). The most differentially expressed region in EBV is shown (bottom of Figure 2C) and interestingly coincides with the loop region of ebv-mir-BART5 which is over 10-fold more frequent in exosomes. The existence of a miRNA-offset RNA (moRNA) in cells can be also observed which is nearly absent in exosomes. As neither loop nor moRNA sequences are generally used as annotations, such findings would be normally missed which shows that the visual exploration of differential expression data can give important information and might trigger or orientate additional analysis. Finally, we analysed the putative functional role of three *Echinococcus granulosus* microRNAs (egr-miR-1, egr-let-7 and egr-miR-8) in the cow host by means of the miRNAfuncTargets workflow. Figure 2D shows the most enriched molecular functions among the cow target genes. It can be seen that many are regulated only by one microRNA but among the most enriched terms are also several that might be regulated by a combination of microRNAs (for example SNAP receptor activity by [egr-miR-1, egr-miR-8]).

## CONCLUSIONS AND FUTURE PLANS

sRNAtoolbox is a flexible and easy to use set of tools for small RNA research. Most tools can be used independently or for downstream analysis of sRNAbench output data. The underlying database contains currently only the most important model species from animals and plants. We are planning to continuously increase the number of species, encouraging all users to request the inclusion of required species. An integrated suite of standalone tools is also in preparation at this moment, which will give users with high workloads the possibility to perform the analysis locally.
